# Research Activity and the Association with Mortality

**DOI:** 10.1371/journal.pone.0118253

**Published:** 2015-02-26

**Authors:** Baris A. Ozdemir, Alan Karthikesalingam, Sidhartha Sinha, Jan D. Poloniecki, Robert J. Hinchliffe, Matt M. Thompson, Jonathan D. Gower, Annette Boaz, Peter J. E. Holt

**Affiliations:** 1 Department of Outcomes Research, St George’s Vascular Institute, London, United Kingdom; 2 NIHR Comprehensive Clinical Research Network Coordinating Centre, Leeds, United Kingdom; 3 Centre for Health and Social Care Research, St George’s University of London, Cranmer Terrace, London, United Kingdom; University of Dundee, UNITED KINGDOM

## Abstract

**Introduction:**

The aims of this study were to describe the key features of acute NHS Trusts with different levels of research activity and to investigate associations between research activity and clinical outcomes.

**Methods:**

National Institute for Health Research (NIHR) Comprehensive Clinical Research Network (CCRN) funding and number of patients recruited to NIHR Clinical Research Network (CRN) portfolio studies for each NHS Trusts were used as markers of research activity. Patient-level data for adult non-elective admissions were extracted from the English Hospital Episode Statistics (2005-10). Risk-adjusted mortality associations between Trust structures, research activity and, clinical outcomes were investigated.

**Results:**

Low mortality Trusts received greater levels of funding and recruited more patients adjusted for size of Trust (n = 35, 2,349 £/bed [95% CI 1,855–2,843], 5.9 patients/bed [2.7–9.0]) than Trusts with expected (n = 63, 1,110 £/bed, [864–1,357] p<0.0001, 2.6 patients/bed [1.7–3.5] p<0.0169) or, high (n = 42, 930 £/bed [683–1,177] p = 0.0001, 1.8 patients/bed [1.4–2.1] p<0.0005) mortality rates. The most research active Trusts were those with more doctors, nurses, critical care beds, operating theatres and, made greater use of radiology. Multifactorial analysis demonstrated better survival in the top funding and patient recruitment tertiles (lowest vs. highest (odds ratio & 95% CI: funding 1.050 [1.033–1.068] p<0.0001, recruitment 1.069 [1.052–1.086] p<0.0001), middle vs. highest (funding 1.040 [1.024–1.055] p<0.0001, recruitment 1.085 [1.070–1.100] p<0.0001).

**Conclusions:**

Research active Trusts appear to have key differences in composition than less research active Trusts. Research active Trusts had lower risk-adjusted mortality for acute admissions, which persisted after adjustment for staffing and other structural factors.

## Introduction

It is widely assumed that patients cared for in research active institutions have better outcomes but evidence for this has been limited. The UK National Institute for Health Research (NIHR) was established in April 2006 to carry forward the vision and goals outlined in the 2005 ‘Best Research for Best Health’ government white paper.[1] The overall mission of the NIHR is to create a health research system in which the NHS supports individuals working in world-class facilities, conducting leading-edge research, focused on the needs of patients and, the public.

One important element in the NIHR’s health research system is the support for, and development of, a national Clinical Research Network (CRN). This provides the infrastructure needed to support both patients and healthcare professionals to participate in research activity. Six NIHR CRNs exist for specific conditions, such as the NIHR Cancer Research Network and the NIHR Primary Care Research Network. The Comprehensive Clinical Research Network (CCRN) provides support for research activity that falls outside the scope of the other networks. Given its wide remit, the CCRN is responsible for distributing most of the NIHR Clinical Research Network funding, equalling 85% of £285m per annum, underpinning research activity across a wide range of disciplines. This translates into both direct research time for clinicians and allied health professionals (nurses, pharmacists, radiographers, laboratory staff etc.) and to research governance support. The level to which the NIHR CCRN supports individual Trusts depends, in the main, on their overall research activity.

Whilst the output of NIHR-funded studies is considerable, there are limited data to suggest a link between research activity and patient outcomes.[2–4] Furthermore, the assessment of any such association is complex.[5,6] In healthcare systems outside the NHS, there is some evidence that the formation of research networks has associations with improved health outcomes. For example, in the USA, an analysis of practice-based research networks (PBRNs) in primary care showed that improved clinical outcomes were apparent for participating practices, and suggested that this was due to a number of factors, each underpinned by infrastructure support from the PBRN.[7] Other studies of primary care and oncology networks have supported this, demonstrating that research networks have “positive and long lasting effects”.[8,9]

Until recently the availability of data on research activity in NHS Trusts was limited. Since 2010 accurate data has been available of NIHR funding and patient recruitment to studies. The aims of this study were to utilise this data to address two questions. First, to investigate whether differences in structural and process factors could be identified between Trusts with varying levels of research activity. Second, to determine whether any association exists between research activity and mortality outcomes from English NHS Trusts for acute admissions.

## Methods

The reporting of this study conforms to the STROBE statement (S1 Appendix).[10]

### Data sources

Three indicators of Trust research activity were utilised, NIHR CCRN funding, number of patients recruited to NIHR CRN portfolio studies and Trust teaching status. To be eligible for inclusion in the portfolio studies must be peer-reviewed, awarded funds through a national competitive process, of discernable value to the NHS and, of high quality.[11] Funding data was restricted to the Comprehensive Clinical Research Network. Patient recruitment to portfolio studies included all Clinical Research Networks (comprehensive, primary care, cancer, diabetes, medicines for children, stroke, dementia and neurodegenerative diseases and mental health networks). Research funding data were provided directly by the NIHR CCRN team from the financial accounts covering the English financial year’s 2010/11.[12] The data comprised the total annual funding given to individual NHS Trusts. Number of patients recruited in 2010/11 to portfolio studies is made publically available by the NIHR annually via The Guardian newspaper website.[13] English NHS Trusts were classified as teaching hospitals if they had a direct and specific link with a member of the Medical School Council in England.[14]

Data on Trust medical staffing numbers, nurse staffing numbers, critical care bed provision, the use of radiological investigations and, operating theatre number were obtained from the Health and Social Care Information Centre (HSCIC).[15–17] Trust operational expense data (as defined by International Financial Reporting Standards) was obtained from trust annual reports. This data was adjusted by the Market Forces Factor which accounts for the particular difficulties of delivering care in the catchment area covered by that Trust.[18]

Patient-level clinical, demographic, and outcomes data were obtained from the Hospital Episode Statistics (HES) 1st April 2005–31st March 2010. The HES is an administrative data warehouse that records the details of every patient admission in England and holds patient-level data on demographics, co-morbidities and, social deprivation indices. A cohort of patients admitted non-electively was taken from a previously published study, and encompassed the breadth of emergency admissions in adults to providers with an acute service.[19] The full detail of case selection and risk models are contained in the prior publication, but, in summary, patient selection was based on primary diagnostic (ICD-10), or procedural code (OPCS-4) (S1 Table). The primary outcome measure was in-hospital death.

As the focus of this study was to examine the relationships between research activity and outcome in acute NHS Trusts, those without emergency departments and specialist institutions, such as rheumatological or women’s hospitals, were excluded. The study was restricted to Trusts in England. Analysis was performed at NHS Trust level (i.e. potentially including more than one physical site) and the term “Trust” is used synonymously with “hospital”.

### Statistical analyses

Analyses were undertaken with SAS version 9.2 (SAS Institute, USA) and R (R Core Team 2013). Trusts were categorised into low mortality, expected mortality and high mortality categories of risk-adjusted inpatient death. Risk adjustment is described previously and used multi-level models incorporating patient demographics, comorbidity and social deprivation scores (based on the patients geographic area of residence) and, a random effects Trust model to account for clustering of outcomes.[19–23] Statistically significant divergence was defined as Trusts outlying the 95% confidence interval of the Poisson distribution and Trusts were subsequently classified as having mortality rates that were high, as expected or, low. Mean CCRN funding and 95% confidence intervals were calculated for each of these three strata. Means were compared with one way ANOVA and Tukey’s range test.[24,25]

Trusts were categorised into tertiles based on their level of NIHR CCRN research funding and separately based on number of patients in NIHR CRN portfolio studies. Associations between these tertiles and mortality was tested in a risk adjusted binary logistic regression model. Odds ratios and 95% confidence intervals were reported and p<0.05 was considered significant.

We used two approaches to determine if the association identified could be explained by the structural or process characteristics of Trusts studied:

1. The unifactorial association of tertile of medical staffing, nurse staffing, critical care beds, radiology utilisation, operating theatres, operational expenses and, teaching status in a risk adjusted binary logistic regression model was identified. Variables with p<0.05 were entered into a multifactorial risk adjusted binary logistic regression model that included tertile of funding and tertile of patient recruitment.
2. K means clustering was undertaken to categorise Trusts according to the availability or utilisation of medical staffing, nurse staffing, critical care beds, radiology utilisation and operating theatres. After re-scaling each variable partitioning around k-medoids was used to determine that English NHS Trusts were clustered into two major groups. The structural details of the two clusters are detailed in Table 1, but can be summarised as trusts characterised by a greater number of doctors, nurses, critical care beds, operating theatres and, radiodiagnostic utilisation (higher resource cluster, n = 23) and those with tendency to less of the same variables (standard resource cluster, n = 117). The unifactorial association of tertile of research funding and tertile of patients recruited was re-analysed within each of these two clusters.

**Table 1:** Structures and processes of NHS trusts as categorised by K means cluster analysis.

	Higher Resourced Cluster	Standard Resourced Cluster
Doctors /bed	0.82 (0.37)	0.58 (0.13)
Nurses /bed	2.4 (0.51)	1.9 (0.30)
Critical care beds /bed	0.039 (0.029)	0.020 (0.007)
Radiodiagnostic procedures /bed	106 (29)	79 (19)
Operating theatres /bed	0.026 (0.004)	0.022 (0.006)

Values in brackets are interquartile ranges.

### Ethics Statement

The HES data contained in this study was obtained with the necessary pre- requisite permissions from the NHS Information Centre (NHS IC). The local ethics board (Wandsworth Research Ethics Committee) has confirmed that formal ethical approval is not required for HES extracts as the data source is pseudo-anonymised and publicly available (upon request). The data was stored on the St George’s University of London secure central server. Although the data are pseudo-anonymised, they fall under the regulation of the Data Protection Act and thus permission has been obtained by the Department of Outcomes Research from the local Caldicott Guardian to store and analyse the data. We did not have direct access to patient notes/records. Informed consent is not obtained from patients included in the study as the data available is pseudo-anonymised and we therefore cannot identify individual patients, though we can track patients through care spells.

## Results

156 trusts were identified of which 140 were suitable for inclusion in the analysis. There was wide variation in the overall NIHR CCRN funding (median £1,297,801, IQR £1,039,015), number of study patients (median 1070, IQR 2300), scaled NIHR CCRN funding (median £1,002/bed, IQR £1,281/bed) and, scaled study patients median (1.8, IQR 2.2) allocated to individual NHS Trusts (Table 2). Trusts in the top tertile of CCRN funding had or utilised greater levels of resource relative to Trust size than Trusts attracting lower levels of CCRN funding (Table 3). Trusts in the top tertile of patient recruitment had greater number of medical and nursing staff as well as critical care beds than trusts with lower levels of recruitment (Table 4).

**Table 2:** Total and scaled NIHR CCRN core research funding and patients recruited to NIHR CRN portfolio studies spread data.

	Minimum	1^st^ Quartile	Median	3^rd^ Quartile	Maximum
CCRN core funding (£)	52,977	379,385	637,926	1,418,400	7,207,747
Scaled CCRN core funding (£/bed)	131	618	1,002	1,899	6,780
Patients recruited CRN portfolio studies	11	628	1070	2928	69260
Scaled patients recruited CRN portfolio studies	0.0139	1.1030	1.795	3.3020	56.1600

Scaled data were calculated £/bed or patient/bed using published Department of Health bed availability data for individual Trusts.

**Table 3:** Trust structures and processes by tertile of CCRN research funding/trust bed.

		Trust NIHR CCRN Funding Level		
		Lowest Tertile of funding (n = 46) (95% confidence interval)	Middle Tertile of funding (n = 47) (95% confidence interval)	Highest Tertile of funding (n = 47) (95% confidence interval)
	Total Trust Beds	687 (592–782)	774 (676–871)	970 (853–1,087)
**Research Measures**	Total CCRN Funding (£)	326,652 (274,920–378,383)	724,159 (623,500–824,818)	2,852,840 (2,306,265–3,399,415)
	CCRN Scaled Funding (£/bed)	507 (463–550)	1,028 (969–1,086)	3,033 (2,610–3,455)
	Teaching hospitals n (%)	7%	6%	38%
**Staffing**	Doctors /bed	0.57 (0.54–0.60) p<0.0001	0.57 (0.54–0.60) p<0.0001	0.79 (0.73–0.85)
	Nurses & HCAs /bed	1.9 (1.83–1.97) p<0.0001	1.83 (1.76–1.90) p<0.0001	2.19 (2.08–2.30)
**Other Structural Measures**	Critical care beds /bed	0.020 (0.018–0.021) p<0.0001	0.022 (0.020–0.024) p<0.0001	0.033 (0.028–0.038)
	Operating Theatres /bed	0.022 (0.021–0.024) p = 0.0629	0.021 (0.020–0.022) p = 0.0011	0.025 (0.023–0.026)
	Radiodiagnostic procedures /bed	83.33 (76.70–89.95) p = 0.0596	81.37 (76.85–85.88) p = 0.0156	92.66 (86.65–98.67)

Tukey’s range test was utilised to identify whether differences between tertiles was statistically significant. P values are for differences with the top tertile. Trusts in the highest tertile of funding consistently had, or used, greater resources than trusts in the middle and lowest tertile. No significant differences between the lowest and middle tertile of funding.

**Table 4:** Trust structures and processes by tertile of patients recruited to NIHR CRN portfolio studies/trust bed.

		Trust Patient Recruitment to NIHR CRN Portfolio Studies		
		Lowest Tertile of patients recruited/bed (n = 46) (95% confidence interval)	Middle Tertile of patients recruited/bed (n = 47) (95% confidence interval)	Highest Tertile of patients recruited/bed (n = 47) (95% confidence interval)
	Total Trust Beds	678 (593–763)	645 (570–719)	890 (780–1001)
**Research Measures**	Patients recruited	543 (449–638)	1218 (1023–1413)	6488 (3577–9398)
	Scaled number of patients recruited (/bed)	0.80 (0.70–0.90)	1.85 (1.73–1.97)	6.87 (4.47–9.26)
	Teaching hospitals n (%)	0%	10%	44%
**Staffing**	Doctors /bed	0.64 (0.61–0.67) p<0.0001	0.69 (0.65–0.72) p<0.0001	0.87 (0.80–0.93)
	Nurses & HCAs /bed	1.7 (1.6–1.7) p<0.0001	1.8 (1.7–1.8) p<0.0001	2.1 (2.0–2.2)
**Other Structural Measures**	Critical care beds /bed	0.021 (0.019–0.023) p = 0.0047	0.021(0.019–0.023) p = 0.0061	0.026 (0.023–0.029)
	Operating Theatres /bed	0.025 (0.024–0.027) p = 0.0990	0.026 (0.024–0.028) p = 0.1928	0.028 (0.026–0.029)
	Radiodiagnostic procedures /bed	108 (101–114) p = 0.0823	120 (112–128) p = 0.9946	119 (111–128)

Tukey’s range test was utilised to identify whether differences between tertiles was statistically significant. P values are for differences with the top tertile. Trusts in the highest tertile of patient recruitment had, or used, greater resources than trusts in the middle and lowest tertile. No significant differences between the lowest and middle tertile of recruitment though for radiodiagnostic procedures p = 0.0656.

### Acute admission analysis

**Fig 1:**
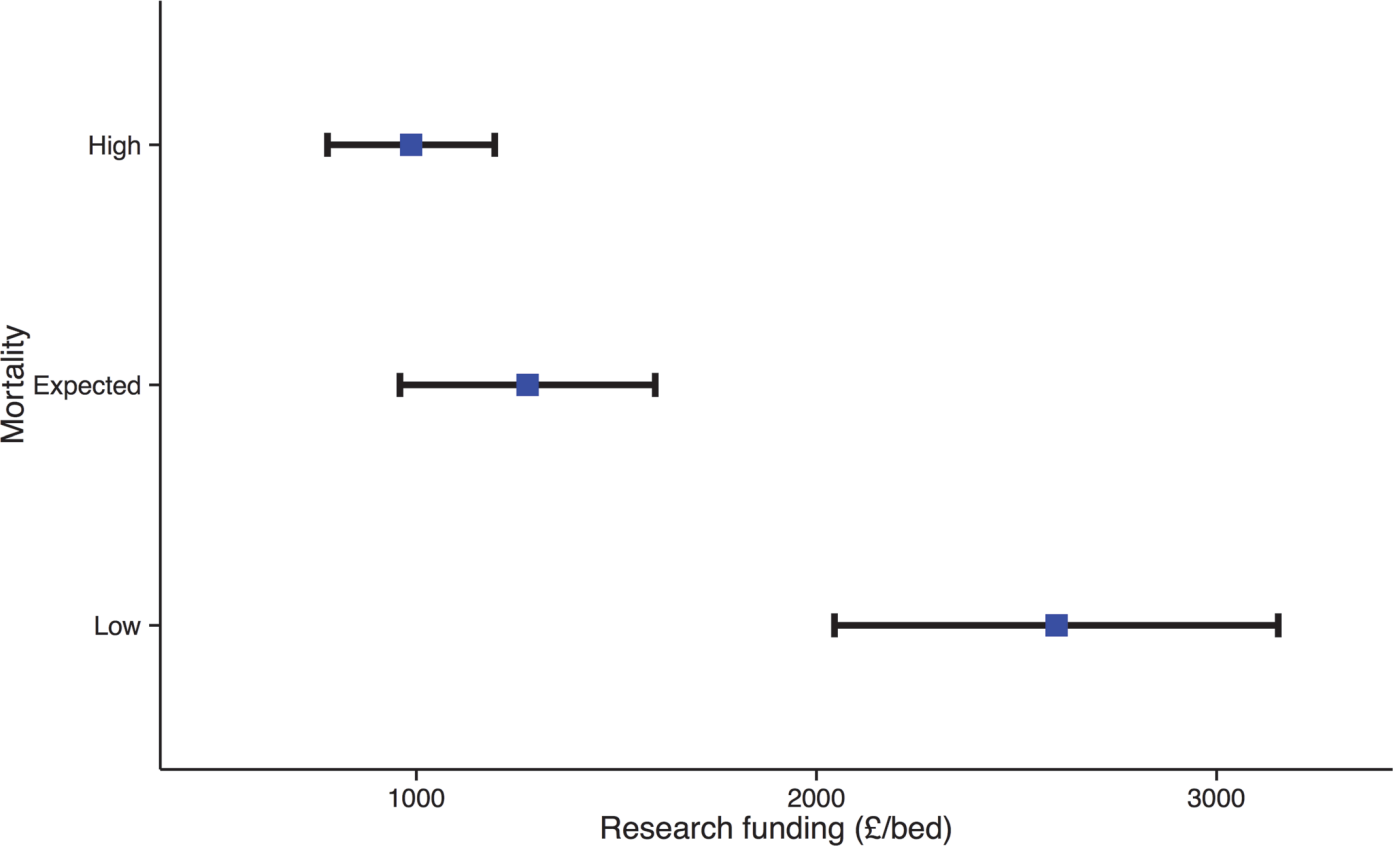
NIHR CCRN funding (£/bed) in English acute NHS Trusts with Trusts sub-grouped as low (n = 35), as expected (n = 63) and, high (n = 42) mortality. For each group, the mean and 95% CI funding are shown. The low mortality Trusts had significantly higher levels of CCRN funding than the as expected (p<0.0001) or high (p = 0.0001) mortality Trusts.

The dataset comprised 2,349,160 adult acute admissions to English NHS trusts in the five-year period. Of these, 1,852,827 were emergency medical admissions and 496,333 emergency surgical admissions. The crude inpatient mortality rate was 15.3% (further demographic details in S2 Table). The total number of patients included is slightly smaller than in our previous study using the same patient definitions, as, due to mergers/closures, 2010/11 research funding data was restricted to fewer trusts. Low mortality outlying Trusts (n = 35, 2600 £/bed, CI 2045–3154) had higher research network funding than Trusts with expected (n = 63,1278 £/bed, CI 959–1597, p<0.0001) or high outlying mortality (n = 42, 987 £/bed, CI 778–1196, p<0.0001) (Fig. 1). Low mortality outlying Trusts (n = 35, 5.9 /bed, CI 2.7–9.0) had more patients recruited than Trusts with expected (n = 63, 2.6 /bed, CI 1.7–3.5, p<0.0169) or high outlying mortality (n = 42, 1.8 /bed, CI 1.4–2.1, p<0.0005).

When Trusts were arranged in tertiles of research funding, patients treated in Trusts in the lowest (1.156 [1.144–1.168] p<0.0001) and middle (1.135 [1.123–1.147] p<0.0001) tertiles had higher odds of death than those in the top tertile (Table 5). Trusts in the lower tertiles of medical staffing, nurse staffing, critical care beds, radiodiagnostic utilisation, operating theatres, operational expenditure and, teaching hospitals also had higher odds ratios for inpatient death (Table 5).

**Table 5:** Unifactorial and multifactorial associations of tertile of Trust research funding, patient recruitment to studies, structures and processes with risk adjusted mortality.

		Unifactorial	Multifactorial
Resource measure by tertile		Odds Ratio of Death (Confidence interval)	Odds Ratio of Death (Confidence interval)
**Research funding**	Lowest	1.156 (1.144–1.168) p<0.0001	1.050 (1.033–1.068) p<0.0001
	Middle	1.135 (1.123–1.147) p<0.0001	1.040 (1.024–1.055) p<0.0001
	Highest	1	1
	Lowest	1.056 (1.043–1.068) p<0.0001	1.069 (1.052–1.086) p<0.0001
**Patients recruited to studies**	Middle	1.056 (1.044–1.069) p<0.0001	1.085 (1.070–1.100) p<0.0001
	Highest	1	1
**Total doctor staffing**	Lowest	1.152 (1.140–1.164) p<0.0001	1.089 (1.069–1.109) p<0.0001
	Middle	1.098 (1.086–1.109) p<0.0001	1.061 (1.047–1.076) p<0.0001
	Highest	1	1
**Total nurse staffing**	Lowest	1.100 (1.089–1.112) p<0.0001	0.960 (0.944–0.975) p<0.0001
	Middle	1.038 (1.028–1.048) p<0.0001	0.985 (0.973–0.998) p = 0.0001
	Highest	1	1
**Total critical care beds**	Lowest	1.060 (1.050–1.072) p<0.0001	0.964 (0.951–0.978) p<0.0001
	Middle	1.065 (1.054–1.076) p<0.0001	1.006 (0.993–1.020) p = 0.3888
	Highest	1	1
**Total radiodiagnostics**	Lowest	1.096 (1.086–1.107) p<0.0001	1.036 (1.022–1.050) p<0.0001
	Middle	1.068 (1.058–1.079) p<0.0001	1.055 (1.043–1.067) p<0.0001
	Highest	1	1
**Operating theatres**	Lowest	1.129 (1.119–1.140) p<0.0001	1.064 (1.049–1.079) p<0.0001
	Middle	1.084 (1.073–1.094) p<0.0001	1.034 (1.022–1.047) p<0.0001
	Highest	1	1
**Operational expenditure**	Lowest	1.158 (1.146–1.170) p<0.0001	1.044 (1.028–1.061) p<0.0001
	Middle	1.093 (1.082–1.104) p<0.0001	1.012 (0.999–1.026) p = 0.0792
	Highest	1	1
**Teaching hospital status**	Non-teaching	1.076 (1.066–1.086) p<0.0001	0.956 (0.942–0.971) p<0.0001
	Teaching	1	1

Of note the association between research funding, patient recruitment and mortality persists in the multifactorial model. The goodness of fit c-statistic (area under the receiver operating characteristic curve) for the multifactorial model is 0.81.

**Fig 2:**
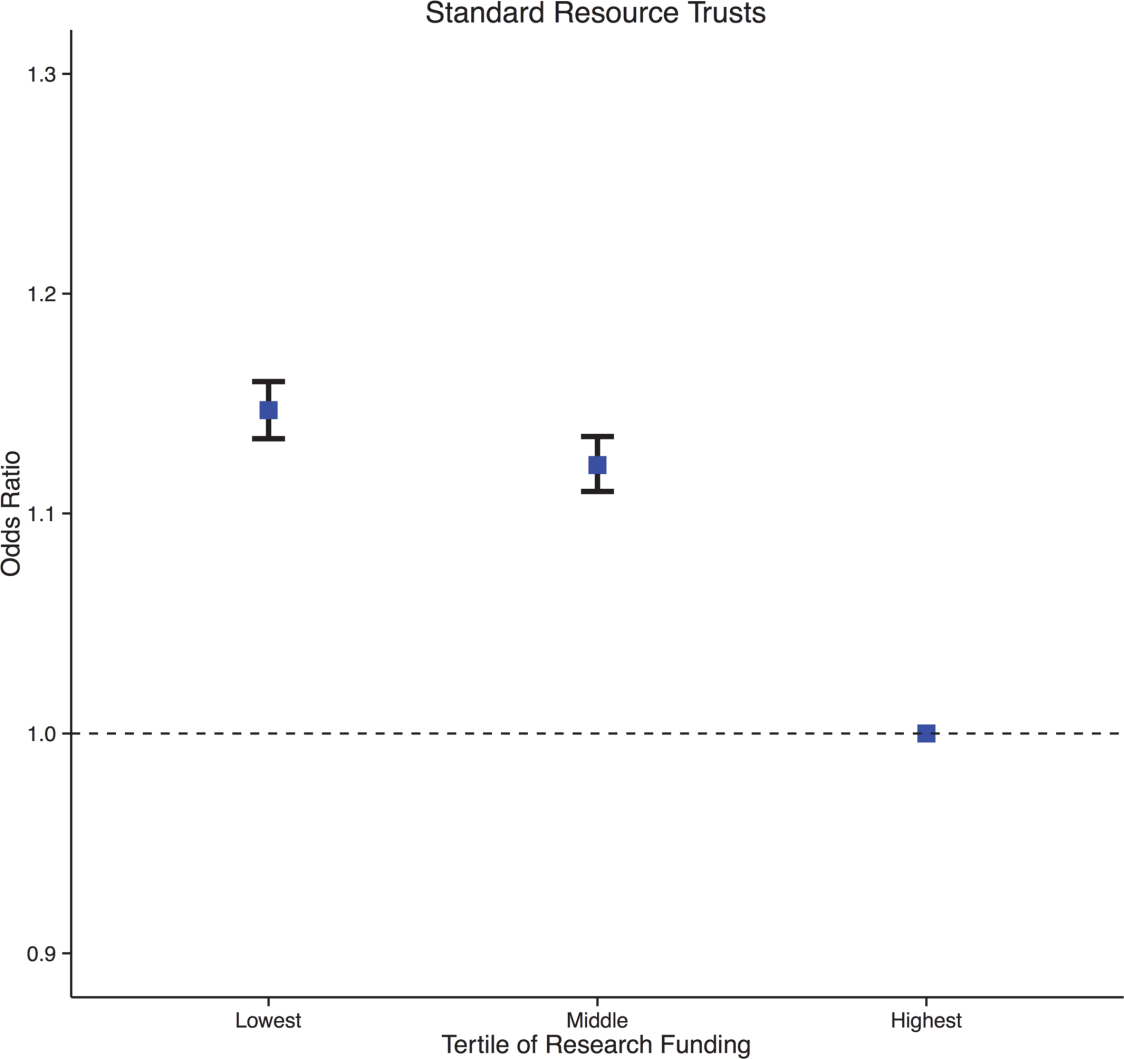
Risk adjusted odds ratio of inpatient death in English NHS Trusts by tertile of scaled CCRN funding. The analysis is restricted to standard resource trusts. For each group, the mean and 95% CI are shown. Trusts in the lowest and middle funding tertile had significantly higher mortality relative to the highest funded trusts.

**Fig 3:**
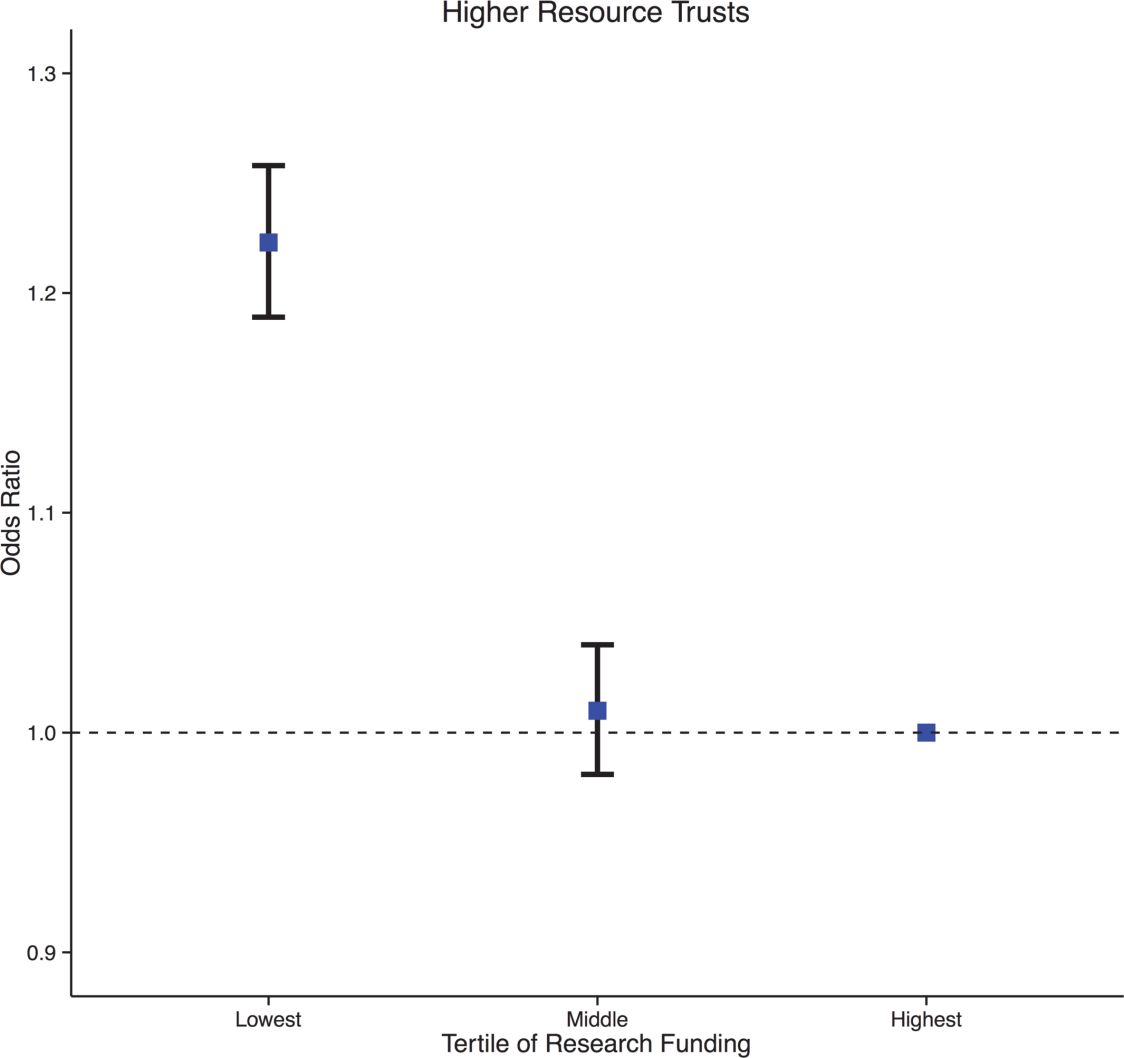
Risk adjusted odds ratio of inpatient death in English NHS Trusts by tertile of scaled CCRN funding. The analysis is restricted to higher resource trusts. For each group, the mean and 95% CI are shown. Trusts in the lowest funding tertile had significantly higher mortality relative to the highest funded trusts.

In multivariate analysis, incorporating research funding, medical staffing, nurse staffing, critical care beds, radiodiagnostic utilisation, operating theatres, operational expenditure and, teaching hospital status, the association between research funding and mortality (lowest versus highest tertile (1.050 [1.033–1.068] p<0.0001), middle versus highest tertile (1.040 [1.024–1.055] p<0.0001)) as well as patient recruitment and mortality persisted (lowest versus highest tertile (1.069 [1.052–1.086] p<0.0001), middle versus highest tertile (1.085 [1.070–1.100] p<0.0001)) (Table 5). The goodness of fit for the multifactorial model was good with a c-statistic (area under the receiver operating characteristic curve) of 0.81.[26] When the analysis was restricted to the standard resource cluster, Trusts in the low (funding 1.147 [1.134–1.160] p<0.0001, recruitment 1.075 [1.063–1.086]) and middle tertiles (funding 1.122 [1.110–1.135] p<0.0001, recruitment 1.072 [1.060–1.083]) of research funding (Fig. 2) and, patients recruited had higher risk-adjusted odds ratios for inpatient death than the highest tertiles. In the high resource cluster, Trusts in the low tertile of research funding (1.223 [1.189–1.258] p<0.0001) (Fig. 3), patient recruitment (1.130 [1.118–1.141]) and, middle tertile of patient recruitment (1.115 [1.104–1.126]) had higher risk-adjusted odds ratios for inpatient death than the highest tertiles.

## Discussion

This study characterises differences in the structures and processes of care between trusts with differential levels of research activity. The most research active Trusts tend, relative to their size to be better staffed in terms of doctors and nurses, have more critical care beds and operating theatres and, have a greater utilisation of advanced radiodiagnostics. Trusts with the best emergency mortality outcomes were those that were most research active. The risk adjusted odds of death was lower in research active Trusts but also Trusts with more of other resources. The association of research activity with outcome could not be explained by staffing, critical care and operating theatre provision or, radiodiagnostic utilisation as the association persisted in the multifactorial models and analysis within clusters of resource.

In multi-level modelling the association of both research funding and number of patients recruited to studies to outcome was independent to teaching hospital status. These findings were consistent with previous work demonstrating no direct relationship between teaching status and healthcare outcomes, but that the number of citations attributable to a trust was correlated with Hospital Standardised Mortality Ratios (HSMR).[27,28]

Whilst there is a plausible link between research engagement and healthcare performance, little empirical evidence currently exists to support this. In particular it has been suggested that research engagement could improve the processes and outcomes of care. These results support this possibility, with research activity being associated with outcomes for acute admissions, rather than being limited only to research participants.

Jarman et al. has previously identified medical staffing to be the best predictor of outcomes for emergency admissions in 1999 but did not include a measure of research activity beyond teaching status.[29] This study demonstrated statistically significant associations independent of teaching status, staffing and, a number of other hospital structures and process.

In terms of understanding these associations, a recent systematic review suggested that engagement with research by individuals and healthcare organisations increased the likelihood of a positive impact on healthcare performance.[4] The number of studies was small, but possible mechanisms for better outcomes included beneficial changes in institutional structure and human capital, specific processes of care related to conducting trials, improvements in organisational mechanisms, and greater levels of collaboration between organisations, teams and, individuals. The review concluded that there was “cumulative evidence that organisations in which the research function is fully integrated into the organisational structure can out-perform other organisations that pay less formal heed to research and its outputs,” which is supported by these findings.

A benefit extending beyond direct research participants has been noted previously, and was reinforced by these pan-provider results. One possible explanation is that some attributes of the setting in which care is delivered, such as equipment and personnel, which are brought in to perform research may remain in place after the research is completed.[30] Furthermore it has been suggested that patients in research active hospitals may have better outcomes than patients in poorly research-active hospitals because greater research participation leads to accumulated knowledge, develops infrastructure and, brings in resources that can be used to improve clinical care.[31,32] This broader impact observed in hospitals with greater network support is supported by three previous studies.[33–35] The suggestion was that hospitals within research networks implement research findings more easily and more quickly, and that clinicians were more likely to adopt evidence-based practice and, follow up-to-date clinical guidelines.

Finally, an American oncological study suggested that greater network involvement was of greater importance than teaching hospital status in the adoption of new and novel treatments, which is commensurate with these results.[34] The reason suggested for this were that research networks act at the interface of research and quality improvement, with networks evolving into learning communities, providing grounds for generalisable solutions to clinical problems.[36,37]

### Strengths and limitations

In terms of determining the reproducibility of these results, a number of different datasets were employed in the analyses, drawn from a number of sources and encompassing different information. The NIHR CCRN funding and recruitment data were provided by the NIHR from their own financial accounts and are accurate for individual trusts. Trust level structure and process data were taken from external datasets, used by the Department of Health for regulatory purposes.

The definition of teaching hospital that we utilised is a traditional one. It should be noted that the majority of district general Trusts in England are involved in teaching both doctors in training and, particularly in recent years increasing numbers of medical students.

We modelled risk adjusted mortality around adult acute admissions over a five-year period, using established methods.[19,38] Risk-standardisation used hierarchical models to account for clustering of deaths, and included widely validated factors.[39–41] The ascertainment of the mortality outcomes from the ONS Registry data adds strength to these results. A further strength of the analysis is the case selection of a group of commonly encountered pathologies ensuring that the link between mortality and quality is plausible as the conditions are amenable to salvage. For example the colorectal laparotomy group only included patients undergoing surgery therefore effectively excluding moribund patients or those with extensive disseminated disease judged to be unfit or inappropriate for surgery. Previous work has identified that mortality metrics calculated using a restricted group of pathologies reduces over-dispersion of data and therefore is superior for comparative statistics.[42] Nevertheless highly specialised or tertiary referral centres often have a high percentage of research active clinical academics and also regional referral intakes with sicker or more complex patients. It is possible therefore that the most physiologically unwell patients (for example in myocardial infarction or pancreatitis) may be cared for in research active units. This would if anything however weaken the association of research activity with better mortality outcomes. It may however explain the unexpected finding in the multifactorial analysis that trusts in the lowest tertile of nurse staffing and critical care provision had more favourable emergency mortality outcomes. Medical staffing and nurse staffing are highly correlated in the English NHS (Spearman rank correlation coefficient of 0.81). Further secondary modelling with an interaction term between medical and nurse staffing demonstrates an interaction but the association with research activity remain (results not shown). It may be that the impact of staffing is complex and needs further separate analysis but this was not pursued, as it is not within the aims of this paper.

We characterised the categories of research funding in terms of a number of hospital structures and processes. We considered the possibility that these were confounders of the association of funding or recruited patient with outcome in our statistical analysis, but the association persisted. The possibility remains however of endogenity due to omitted co-factors. Future research will need to assess further the interaction of infrastructure with research activity. The aim of our study however was not to prove causality between funding or recruited patient number with outcome. Both were used as surrogates for research activity. Although we controlled for a number of important factors it seems likely that other unmeasured confounders (resources) could influence outcome. Inverse causality is also a possibility wherein better-resourced hospitals with better outcomes attract research funding and therefore research active. It is of note however that in our sensitivity analysis research funding and recruitment were associated with outcome even in the higher resource cluster of trusts. There may be organisational, administrative or managerial factors which impact on a Trusts ability to gain research funding, recruit patients to studies as well as patient care. This could therefore confound the relationship between research activity and outcome. Accurate funding and patient recruitment data prior to 2010/2011 was not available. Future research should study longitudinal trends in research activity, potential confounders and outcome. Such an analysis would better help to understand the mechanisms (whether causal or confounded) behind the association of research activity with outcome.

This work was limited by the use of only a single measure of research funding, NIHR CCRN core funding, and a focus on mortality as an outcome. Determining the association of the largest national funding stream on a hard, validated outcome measure would appear an intuitive starting position in a detailed examination of measuring the success of national research infrastructure. Future research should include measures of morbidity and patient reported outcome measures. More granular information with details of multiple funding streams would provide a clearer picture of how and where the greatest gains in outcome have been made. It was not possible to include a measure of research funding by industry, which can account for a significant proportion of activity in some trusts, as such data is not readily available in the United Kingdom. It is therefore plausible that the associations observed are secondary to alternative research funding streams. This would not however detract from the observation that research activity associates with clinical outcomes. Future research should investigate whether the type of studies to which Trusts recruit (interventional versus observational) is associated with differences in outcome. Accurate data for the type of study was not available for 2010/2011 but is now being collected and being made publically available. In addition, non-mortality outcomes should be investigated where data exist on a national scale.

## Conclusions

This study provides evidence that research activity in acute English NHS Trusts is associated with lower mortality outcomes for emergency admissions. The reasons for this is not fully established. These results should act as a catalyst to understand the relationships in more detail with scope for further quantitative analyses.

## Supporting Information

S1 Appendix Strobe Statement. (DOC)

S1 Table OPCS-4 codes used to define emergency medical conditions and emergency surgical procedures. *NEC = not elsewhere classified, **EC = elsewhere classified.
(DOC)

S2 Table Demographic data and crude inpatient mortality outcomes for acute admissions. (DOC)
