# Supplementary material for: Research Activity and the Association with Mortality
Source: PLoS One. 2015 Feb 26;10(2):e0118253. doi: 10.1371/journal.pone.0118253 (PMC4342017; doi:10.1371/journal.pone.0118253)
Supplement: S1 Appendix — (DOC) [file pone.0118253.s001.doc]

|  | Item No | | Recommendation |
| --- | --- | --- | --- |
| **Title and abstract** | 1 | | *(*a) Indicate the study’s design with a commonly used term in the title or the abstract  Page 2 |
|  |  |  | (*b*) Provide in the abstract an informative and balanced summary of what was done and what was found  Page 2 |
| Introduction | | | |
| Background/rationale | 2 | Explain the scientific background and rationale for the investigation being reported  Pages 3 and 4 | |
| Objectives | 3 | State specific objectives, including any prespecified hypotheses  Page 4 | |
| Methods | | | |
| Study design | 4 | Present key elements of study design early in the paper  Pages 4 to 6 | |
| Setting | 5 | Describe the setting, locations, and relevant dates, including periods of recruitment, exposure, follow-up, and data collection  Pages 4 and 5 | |
| Participants | 6 | *(*a) Give the eligibility criteria, and the sources and methods of selection of participants. Describe methods of follow-up  Page 5 | |
|  |  | (*b*) For matched studies, give matching criteria and number of exposed and unexposed  Not applicable | |
| Variables | 7 | Clearly define all outcomes, exposures, predictors, potential confounders, and effect modifiers. Give diagnostic criteria, if applicable.  Pages 4 to 6 and Appendix | |
| Data sources/ measurement | 8* | For each variable of interest, give sources of data and details of methods of assessment (measurement). Describe comparability of assessment methods if there is more than one group.  Pages 4 to 6 | |
| Bias | 9 | Describe any efforts to address potential sources of bias  Page 6 | |
| Study size | 10 | Explain how the study size was arrived at  Not applicable | |
| Quantitative variables | 11 | Explain how quantitative variables were handled in the analyses. If applicable, describe which groupings were chosen and why  Pages 4-6 | |
| Statistical methods | 12 | *(*a) Describe all statistical methods, including those used to control for confounding  Pages 5 and 6 | |
|  |  | (*b*) Describe any methods used to examine subgroups and interactions  Page 5 and 6 | |
|  |  | (*c*) Explain how missing data were addressed  Page 5 and reference 18 | |
|  |  | (*d*) If applicable, explain how loss to follow-up was addressed  Not applicable | |
|  |  | (*e*) Describe any sensitivity analyses  Not applicable | |
| Results | | | |
| Participants | 13* | (a) Report numbers of individuals at each stage of study—eg numbers potentially eligible, examined for eligibility, confirmed eligible, included in the study, completing follow-up, and analysed  Not applicable – only eligible subjects were extracted from the database. All eligible subjects (barring those with missing data) were subsequently included in analyses. Appendix for demographic data | |
|  |  | (b) Give reasons for non-participation at each stage  Not applicable | |
|  |  | (c) Consider use of a flow diagram  Not applicable | |
| Descriptive data | 14* | (a) Give characteristics of study participants (eg demographic, clinical, social) and information on exposures and potential confounders  Appendix and reference 18 | |
|  |  | (b) Indicate number of participants with missing data for each variable of interest  Reference 18 | |
|  |  | (c) Summarise follow-up time (eg average and total amount)  One year for all patients | |
| Outcome data | 15* | Report numbers of outcome events or summary measures over time  Page 7 | |
| Main results | 16 | *(*a) Give unadjusted estimates and, if applicable, confounder-adjusted estimates and their precision (eg, 95% confidence interval). Make clear which confounders were adjusted for and why they were included.  Crude outcomes data listed in Appendix. | |
|  |  | (*b*) Report category boundaries when continuous variables were categorized  Table 3 and 4 | |
|  |  | (*c*) If relevant, consider translating estimates of relative risk into absolute risk for a meaningful time period  Not applicable | |
| Other analyses | 17 | Report other analyses done—eg analyses of subgroups and interactions, and sensitivity analyses  Page 8 | |
| Discussion | | | |
| Key results | 18 | Summarise key results with reference to study objectives  Pages 8 and 9 | |
| Limitations | 19 | Discuss limitations of the study, taking into account sources of potential bias or imprecision. Discuss both direction and magnitude of any potential bias.  Page 11 | |
| Interpretation | 20 | Give a cautious overall interpretation of results considering objectives, limitations, multiplicity of analyses, results from similar studies, and other relevant evidence.  Pages 9 to 11 | |
| Generalisability | 21 | Discuss the generalisability (external validity) of the study results.  Page 12 | |
| Other information | | | |
| Funding | 22 | Give the source of funding and the role of the funders for the present study and, if applicable, for the original study on which the present article is based  Pages 7 and 13 | |

*Give information separately for exposed and unexposed groups.

**Note:** An Explanation and Elaboration article discusses each checklist item and gives methodological background and published examples of transparent reporting. The STROBE checklist is best used in conjunction with this article (freely available on the Web sites of PLoS Medicine at http://www.plosmedicine.org/, Annals of Internal Medicine at http://www.annals.org/, and Epidemiology at http://www.epidem.com/). Information on the STROBE Initiative is available at http://www.strobe-statement.org.
