# Supplementary material for: Research Activity and the Association with Mortality
Source: PLoS One. 2015 Feb 26;10(2):e0118253. doi: 10.1371/journal.pone.0118253 (PMC4342017; doi:10.1371/journal.pone.0118253)
Supplement: S1 Table — *NEC = not elsewhere classified, **EC = elsewhere classified. (DOC) [file pone.0118253.s002.doc]

| **Emergency medical condition/surgical procedure** | **ICD-10 codes (medical)/OPCS-4 codes (surgical)** | **Additional logic** |
| --- | --- | --- |
| Acute myocardial infarction | I21 Acute myocardial infarction  I22 Subsequent myocardial infarction | Emergency mode of admission, primary diagnosis only |
| Congestive cardiac failure | I50 Heart failure  I110 Hypertensive heart disease with heart failure | Emergency mode of admission, primary diagnosis only |
| Stroke | I61 Intracerebral haemorrhage  I63 Cerebral infarction  I64 Stroke, not specified as haemorrhage or infarction | Emergency mode of admission, primary diagnosis only |
| Pneumonia | J10 Influenza due to other identified influenza virus  J11 Influenza, virus not identified  J12 Viral pneumonia NEC*  J13 Pneumonia due to Streptococcus pneumonia  J14 Pneumonia due to Haemophilus influenzae  J15 Bacterial pneumonia NEC*  J16 Pneumonia due to other organisms NEC*  J17 Pneumonia in bacterial diseases EC**  J18 Pneumonia, organism unspecified | Emergency mode of admission, primary diagnosis only |
| Pulmonary embolism | I26 Pulmonary embolism | Emergency mode of admission, primary diagnosis only |
| Urinary tract infection | N390 Urinary tract infection, site not specified  N30 Cystitis | Emergency mode of admission, primary diagnosis only |
| Sepsis | A40 Streptococcal sepsis  A41 Other sepsis  A392-A394 Meningococcaemia  R508 Other specified fever  R509 Fever, unspecified  R572 Septic shock  R578 Other shock  R579 Shock, unspecified | Emergency mode of admission, primary diagnosis only |
| Cardiac Arrest | I46 Cardiac arrest | Emergency mode of admission, primary diagnosis only |
| Pancreatitis | K85 Acute pancreatitis | Emergency mode of admission, primary diagnosis only |
| Repair of fractured neck of femur | W191 Primary open reduction of fracture of neck of femur and open fixation using pin and plate  W241 Closed reduction of intracapsular fracture of neck of femur and fixation using nail or screw  W461 Primary prosthetic replacement of head of femur using cement  W468 Other specified prosthetic replacement of head of femur using cement  W469 Unspecified prosthetic replacement of head of femur using cement  W471 Primary prosthetic replacement of head of femur not using cement  W478 Other specified prosthetic replacement of head of femur not using cement  W479 Unspecified prosthetic replacement of head of femur not using cement  W481 Primary prosthetic replacement of head of femur NEC*  W488 Other specified other prosthetic replacement of head of femur  W489 Unspecified other prosthetic replacement of head of femur | Emergency mode of admission, primary procedure only |
| Emergency hernia surgery | T97 Repair of recurrent umbilical hernia  T20 Repair of primary inguinal hernia  T21 Repair of recurrent inguinal hernia  T22 Repair of primary femoral hernia  T23 Repair of recurrent femoral hernia  T24 Repair of umbilical hernia  T25 Repair of incisional hernia  T26 Repair of recurrent incisional hernia  T27 Repair of ventral hernia | Emergency mode of admission, primary procedure only |
| Emergency appendicectomy | H01 Emergency excision of appendix  H028 Other specified other excision of appendix  H029 Unspecified other excision of appendix  H03 Drainage of abscess of appendix | Emergency mode of admission, primary procedure only |
| Emergency peptic ulcer surgery | G351 Closure of perforated ulcer of stomach  G352 Closure of ulcer of stomach NEC  G358 Other specified operations on ulcer of stomach  G359 Unspecified operations on ulcer of stomach  G362 Closure of perforation of stomach NEC  G412 Repair of perforation of pylorus  G521 Closure of perforated ulcer of duodenum  G522 Suture of ulcer of duodenum NEC  G523 Oversew of blood vessel of duodenal ulcer  G528 Other specified operations on ulcer of duodenum  G529 Unspecified operations on ulcer of duodenum  G532 Closure of perforation of duodenum NEC | Emergency mode of admission, primary procedure only |
| Emergency colorectal laparotomy | H041 Panproctocolectomy and ileostomy  H05 Total colectomy  H06 Extended right hemicolectomy  H07 Right hemicolectomy  H08 Transverse colectomy  H09 Left hemicolectomy  H10 Sigmoid colectomy  H11 Colectomy NEC*  H12 Excision of lesion of colon  H13 Bypass of colon  H141-142, H144 Caecostomy  H151-153 Colostomy  H158-159 Exteriorisation of colon  H16 Caecotomy/colotomy  H17 Open reduction of colonic intussusception/volvulus/obstruction NEC*  H33 APER/anterior resection/excision of rectum  G713-715 Bypass of ileum by anastomosis of ileum to colon  G721-725 Anastomosis of ileum to colon/rectum/anus  G734 Resection of ileo-colic anastomosis | Emergency mode of admission, primary procedure only |
| Emergency urological surgery | M021 Nephrectomy and excision of perirenal tissue  M022 Nephroureterectomy NEC  M023 Bilateral nephrectomy  M024 Excision of half of horseshoe kidney  M025 Nephrectomy NEC  M028 Other specified total excision of kidney  M029 Unspecified total excision of kidney  M03 Partial excision of kidney  M04 Deroofing of cyst/destruction of kidney lesion  M051 Open pyeloplasty  M052 Open revision of pyeloplasty  M18 Excision of ureter  M34 Cystectomy  M35 Partial excision of bladder  M61 Prostatectomy  M058 Other specified open repair of kidney  M059 Unspecified open repair of kidney  M06 Open removal of renal calculus/drainage of kidney  M053 Nephropexy  M09 Endoscopic fragmentation/extraction of calculus of kidney  M10 Endoscopic destruction of lesion of kidney/pyeloplasty/deroofing of cyst  M13 Percutaneous biopsy/drainage/aspiration/injection of substance into kidney  M14 Extracorporeal fragmentation of calculus of kidney  M15 Nephrostomography/other specified or unspecified operations on kidney along nephrostomy tube track  M16 Irrigation of kidney/percutaneous nephrolithotomy NEC/removal of nephrostomy tube/other specified or unspecified operations on kidney  M27 Ureteroscopic fragmentation/extraction of calculus of ureter/insertion of ureteric stent/dilation of ureter  M28 Endoscopic fragmentation/removal of calculus of ureter  M29 Endoscopic insertion/removal of prosthesis into ureter (NEC)/extirpation of lesion of ureter/dilation of ureter  M21 Anastomosis of ureter to bladder/ureter/reconstruction of ureter  M22 Repair of ureter  M26 Nephroscopic fragmentation/extraction of calculus or ureter/insertion of prosthesis  M33 Percutaneous insertion of stent into ureter  M31 Extracorporeal fragmentation of calculus of ureter  M37 Cystourethroplasty/repair of bladder/vesicocolic fistula/other specified or unspecified repair of bladder  M38 Cystostomy/other specified or unspecified open drainage of bladder  M42 Endoscopic destruction of lesion of bladder  M44 Endoscopic removal of calculus/blood clot/foreign body from bladder/other specified or unspecified endoscopic operations on bladder | Emergency mode of admission, primary procedure only |
| Emergency open repair of infra-renal abdominal aortic aneurysm | L184-189 Emergency replacement of aneurysmal segment of infra-renal abdominal aorta  L194-199 Replacement of aneurysmal segment of infra-renal abdominal aorta  L231, L236, L238-239 Plastic repair of aorta  L254, L258, L259 Operations on aortic aneurysm NEC*  L49 Replacement of aneurysmal iliac artery | Elective or emergency operation codes in conjunction with emergency mode of admission |
| Emergency endovascular repair of infra-renal abdominal aortic aneurysm | L265 Percutaneous transluminal insertion of stent into aorta  L271 Endovascular insertion of stent graft for infra-renal abdominal aortic aneurysm  L275 Endovascular insertion of stent graft for aortic aneurysm of bifurcation NEC  L276 Endovascular insertion of stent graft for aorto-uniiliac aneurysm  L278 Other specified transluminal insertion of stent graft for aneurysmal segment of aorta  L279 Unspecified transluminal insertion of stent graft for aneurysmal segment of aorta  L281 Endovascular stenting for infra-renal abdominal aortic aneurysm  L285 Endovascular stenting for aortic aneurysm of bifurcation NEC  L286 Endovascular stenting for aorto-uniiliac aneurysm  L289 Unspecified transluminal operations on aneurysmal segment of aorta  Any elective or emergency open AAA code in conjunction with (Y022 [other specified other endovascular placement of stent] and Z346 Z347 Z348 Z349 [abdominal aorta, infra-renal abdominal aorta, aorta NEC]), (O203 O204 O205 O208 O209 [endovascular placement of one or more stent grafts] and Y528 Y53 Y76.9 Y78 [other specified approach to organ through other opening, approach to organ under radiological control, unspecified minimal access to other body cavity, arteriotomy approach to organ using radiological guidance]), (L281 L285 L286 L289) or (L761 L762 L765 L768) | Elective or emergency operation codes in conjunction with emergency mode of admission |
| Emergency carotid endarterectomy | L294 Endarterectomy of carotid artery and patch repair of carotid artery  L295 Endarterectomy of carotid artery NEC  L298 Other specified reconstruction of carotid artery  L299 Unspecified reconstruction of carotid artery | Emergency mode of admission |
| Emergency lower extremity arterial revascularisation | L16 Axillo-femoral bypass/other extra-anatomic aortic bypass  L206 Emergency aorto-iliac bypass  L216 Aorto-iliac bypass  L50 Emergency iliac bypass  L51 Iliac bypass  L52 Endarterectomy of iliac artery  L652-653 Revision of iliac/femoral artery reconstruction  L62 Repair/embolectomy of femoral artery  L58 Emergency femoro-distal bypass  L59 Femoro-distal bypass  L60 Endarterectomy/profundoplasty of femoral artery | Elective or emergency operation codes in conjunction with emergency mode of admission |
| Emergency lower extremity amputation | X093 Amputation of leg above knee  X094 Amputation of leg through knee  X095 Amputation of leg below knee  X098 Other specified amputation of leg  X099 Unspecified amputation of leg  X10 Amputation of foot  X11 Amputation of toe  X12 Revision of amputation | Emergency mode of admission |
