# Supplementary material for: Research Activity and the Association with Mortality
Source: PLoS One. 2015 Feb 26;10(2):e0118253. doi: 10.1371/journal.pone.0118253 (PMC4342017; doi:10.1371/journal.pone.0118253)
Supplement: S2 Table — (DOC) [file pone.0118253.s003.doc]

| Diagnosis/procedure | All Acute Admissions |
| --- | --- |
| Number of cases/procedures | 2,349,160 |
| Number of hospitals | 156 |
| Mean patient age (years) | 69 |
| Male gender (%) | 47.8 |
| RCS Charlson co-morbidity score (%) | 0(34.7) 1(34.5) 2(19.1) 3(11.7) |
| Social deprivation quintile (%) | 1(22.5) 2(20.9) 3(20.2) 4(19.2) 5(17.1) |
| Number of deaths | 358,684 |
| In-hospital mortality rate (%) | 15.3 |
